# Short-term effects of neuromuscular blockade on global and regional lung mechanics, oxygenation and ventilation in pediatric acute hypoxemic respiratory failure

**DOI:** 10.1186/s13613-016-0206-9

**Published:** 2016-10-26

**Authors:** Marlon E. F. Wilsterman, Pauline de Jager, Robert Blokpoel, Inez Frerichs, Sandra K. Dijkstra, Marcel J. I. J. Albers, Johannes G. M. Burgerhof, Dick G. Markhorst, Martin C. J. Kneyber

**Affiliations:** 1 Division of Paediatric Intensive Care, Department of Paediatrics, Beatrix Children’s Hospital, University Medical Center Groningen, University of Groningen, P.O. Box 30.001, 9700 RB Groningen, The Netherlands; 2 Department of Paediatrics, Nij Smellinghe Hospital, Drachten, The Netherlands; 3 Department of Anaesthesiology and Intensive Care Medicine, University Medical Center Schleswig-Holstein, Campus Kiel, Kiel, Germany; 4 Department of Epidemiology, University Medical Center Groningen, University of Groningen, Groningen, The Netherlands; 5 Division of Paediatric Intensive Care, Department of Paediatrics, VU University Medical Center, Amsterdam, The Netherlands; 6 Critical Care, Anaesthesia, Peri-operative Medicine and Emergency Medicine (CAPE), University of Groningen, Groningen, The Netherlands

**Keywords:** Acute hypoxemic respiratory failure, Pediatric acute respiratory distress syndrome, Neuromuscular blockade, Electrical impedance tomography, Lung mechanics, Oxygenation, Mechanical ventilation, Children

## Abstract

**Background:**

Neuromuscular blockade (NMB) has been shown to improve outcome in acute respiratory distress syndrome (ARDS) in adults, challenging maintaining spontaneous breathing when there is severe lung injury. We tested in a prospective physiological study the hypothesis that continuous administration of NMB agents in mechanically ventilated children with severe acute hypoxemic respiratory failure (AHRF) improves the oxygenation index without a redistribution of tidal volume *V*_T_ toward non-dependent lung zones.

**Methods:**

Oxygenation index, PaO_2_/FiO_2_ ratio, lung mechanics (plateau pressure, mean airway pressure, respiratory system compliance and resistance), hemodynamics (heart rate, central venous and arterial blood pressures), oxygenation [oxygenation index (OI), PaO_2_/FiO_2_ and SpO_2_/FiO_2_], ventilation (physiological dead space-to-*V*_T_ ratio) and electrical impedance tomography measured changes in end-expiratory lung volume (EELV), and *V*_T_ distribution was measured before and 15 min after the start of continuous infusion of rocuronium 1 mg/kg. Patients were ventilated in a time-cycled, pressure-limited mode with pre-set *V*_T_. All ventilator settings were not changed during the study.

**Results:**

Twenty-two patients were studied (*N* = 18 met the criteria for pediatric ARDS). Median age (25–75 interquartile range) was 15 (7.8–77.5) weeks. Pulmonary pathology was present in 77.3%. The median lung injury score was 9 (8–10). The overall median CoV and regional lung filling characteristics were not affected by NMB, indicating no ventilation shift toward the non-dependent lung zones. Regional analysis showed a homogeneous time course of lung inflation during inspiration, indicating no tendency to atelectasis after the introduction of NMB. NMB decreased the mean airway pressure (*p* = 0.039) and OI (*p* = 0.039) in all patients. There were no significant changes in lung mechanics, hemodynamics and EELV. Subgroup analysis showed that OI decreased (*p* = 0.01) and PaO_2_/FiO_2_ increased (*p* = 0.02) in patients with moderate or severe PARDS.

**Conclusions:**

NMB resulted in an improved oxygenation index in pediatric patients with AHRF. Distribution of *V*_T_ and regional lung filling characteristics were not affected.

## Background

Severe respiratory failure ranging from acute hypoxemic respiratory failure (AHRF) to acute respiratory distress syndrome (ARDS) is associated with substantial morbidity and mortality in up to 30–50% of critically ill children [1, 2]. So far, management of these children has been confined to a lung-protective mechanical ventilation (MV) strategy entailing low tidal volume (*V*_T_) of 6 mL/kg body weight, limiting peak inspiratory pressure (PIP) and/or plateau pressure (*P*_plat_) to 30 cmH_2_O and the application of positive end-expiratory pressure (PEEP) [3, 4].

During spontaneous ventilation, tidal breathing is directed to the dependent, well-perfused regions of the lung as opposed to during neuromuscular blockade (NMB) [5]. Hence, maintaining spontaneous breathing has been advocated for patients with ARDS as it reduces shunt and decreases dead space [6–8]. However, there may be a role for NMB agents when there is severe lung injury. Papazian et al. have shown in the *ARDS et Curarisation Systematique* (ACURASYS) trial that 48-h use of NMB agents in *N* = 340 adults with early severe ARDS (i.e., PaO_2_/FiO_2_ < 150 mmHg and PEEP > 5 cmH_2_O) significantly reduced the 90-day crude mortality from 40.7% (95% confidence interval 33.5–48.4) in the placebo group to 31.6% (95% CI 25.2–38.8) in the cisatracurium group [9]. No significant side effects including ICU-acquired weakness were observed in this study. These findings remained true when combined with earlier smaller studies from the same group of investigators in a meta-analysis [10]. The pathophysiological mechanisms underlying the beneficial effects of short-term use of NMB agents in severe ARDS remain speculative but are most likely multifactorial including a minimization of barotrauma, reduced patient-ventilator asynchrony, prevention of active expiration, decreased lung inflammation and reduced oxygen consumption (${\dot{\text{V}}}$O_2_) resulting from a decreased work of breathing (WOB) [11–13]. Also, Yoshida et al. showed the occurrence of pendelluft toward the dependent zones during spontaneous breathing, thereby leading to local overstretch of the dependent lung zones [14].

NMB agents are used in a variable proportion of mechanically ventilated children to facilitate MV [15, 16]. However, it is unclear whether the effects of NMB agents on *V*_T_ distribution and pendelluft also occur in mechanically ventilated children with severe AHRF. The objectives of this study were to test global and regional *V*_T_ distribution and lung mechanics in mechanically ventilated children with severe AHRF before and 15 min after start of continued use of NMB agents.

## Methods

### Study design

This study was designed as a prospective physiological study. It was performed in the Pediatric Intensive Care Unit of the Beatrix Children’s Hospital, University Medical Center Groningen (Groningen, the Netherlands). The institutional review board of the hospital approved the study. Patients were enrolled after getting written informed consent from either parents or legal caretakers.

### Patients

Mechanically ventilated children younger than 12 years with AHRF (defined by acute onset, presence of ≥1 (bilateral) infiltrates on chest radiograph, PaO_2_/FiO_2_ < 300 mmHg and PEEP ≥ 5 cmH_2_O) were included after the attending physician set the indication for NMB within the first 48 h after onset of AHRF. Continuous use NMB in patients with AHRF is usually started if the peak inspiratory pressures approximate 28–30 cmH_2_O, and the patient is hypoxemic and continues to show excessive WOB despite adequate sedation and/or analgesia. Train-of-four to assess efficacy of NMB was not available. Severity of AHRF was quantified using the pediatric modification of the Murray Lung Injury Score (LIS) [17]. ARDS was defined by the pediatric ARDS (PARDS) definition [18]. Prematurely born children with a gestational age corrected for postconceptional age <40 weeks were excluded because we did not want to include perinatal related disease as cause of ARDS, congenital or acquired neuromuscular disorders because of the potential contraindication for neuromuscular blocking agents, severe traumatic brain injury (i.e., Glasgow Coma Score < 8) because of strict* p*CO_2_ management, congenital or acquired paralysis of the diaphragm because they cannot sufficiently breathe spontaneously before start of neuromuscular blocking agents, uncorrected congenital heart disorder when there was a mixed circulation, thereby affecting metric of oxygenation and severe pulmonary hypertension because strict *p*O_2_ and *p*H management were excluded.

### Ventilator protocol

All patients were in the supine position and received continuous intravenous infusion of analgo-sedative drugs. Dosage and targets were uniform for all patients and were not changed during the study. Patients were ventilated in a time-cycled, pressure-limited mode with pre-set *V*_T_ (i.e., pressure-regulated volume-controlled ventilation) in the synchronized intermittent mandatory ventilation (SIMV) setting defined by the attending physician in agreement with the local guideline [entailing peak inspiratory pressure (PIP) < 30–35 cmH_2_O and *V*_T_ 6–8 mL/kg actual body weight, pressure support equals measured PIP minus PEEP]. Ventilator settings including mandatory breath rate, inspiratory time, *V*_T_, positive end-expiratory pressure (PEEP) and FiO_2_ remained constant throughout the study period. Recruitment maneuvers were not performed in any patient at any time point.

### Measurements

Measurements were taken prior to (M0) and 15 min after (M1) a bolus of 1 mg/kg rocuronium bromide and subsequent continuous intravenous infusion of 1 mg/kg/h. Blood samples were drawn from the radial or femoral arteries to determine arterial partial pressures of CO_2_ (PaCO_2_) and O_2_ (PaO_2_). *P*_plat_ and mean airway pressure (mPaw) and respiratory system compliance (Crs) were obtained from the ventilator (Evita XL, Drager, Lubeck, Germany) after a 3-s inspiratory hold in agreement with the manufacturer’s manual. End-tidal CO_2_ was measured using the mainstream capnography sensor and cuvette according to the manufacturer’s manual. Dead space-to-tidal volume ratio was assessed by calculating the end-tidal alveolar dead space fraction (AVDSF) which has shown to be a good proxy [19].

Electrical impedance tomography (EIT) was used to examine the distribution of regional *V*_T_ and the homogeneity of regional lung inflation using the Goe-MF II EIT system (CareFusion, Yorba Linda, CA, USA) as described previously [20, 21]. Additionally, the changes in end-expiratory lung volume (EELV) were also determined using the EIT data [22–24]. The general principle of EIT has been explained elsewhere in detail [25, 26]. Briefly, 16 X-ray translucent electrodes (Blue Sensor BR-50-K, Ambu, Denmark) were applied around the patient’s chest in one transverse plane at the level of the intermammary line. One-minute recordings were made at a scan rate of 13 Hz. The resulting raw EIT images, representing relative impedance changes (Δ*Z*) in the chest cross section with a spatial resolution of 32 × 32 image pixels, were calculated using a 30-s reference period just before M0. The series of raw images were analyzed offline.

### Offline analysis of EIT data

The EIT data were low-pass filtered with a cutoff frequency of 2 Hz to eliminate small impedance changes synchronous with the heartbeat. The threshold tidal impedance variation limit to define the lung regions in EIT images was set at 20% of the maximum variation [27]. Further analysis was confined to these lung regions.

At first, functional EIT images were generated from the acquired series of raw EIT images by calculating the average tidal variation of Δ*Z* in each image pixel. These tidal functional EIT images represented the distribution of *V*_T_ in the studied chest section. In the next step, the distribution of *V*_T_ was characterized with respect to its most relevant, anteroposterior direction by the center of ventilation (CoV). CoV was calculated as described previously [20]. Briefly, CoV location is given in relation to the anteroposterior chest diameter. Values lower than 50% occur when CoV is located in the anterior half of the chest, i.e., ventilation is directed toward non-dependent regions in supine subjects. Values higher than 50% imply predominant ventilation of dependent regions.

Regional filling characteristics of the lungs were calculated in agreement with previous reports [21, 28]. In aggregate, pixel tidal inspiration EIT waveforms were plotted versus the global waveforms calculated from the sum of all image pixels. The data were normalized to the individual pixel and global maximum tidal difference in relative Δ*Z* (thus, they are given as fractions of 1.0). These plots were then fitted by a polynomial function of the second degree. Fittings were accepted if the correlation coefficient (*R* ^2^) was ≥0.90. Generally, the higher the absolute value of the pixel polynomial coefficient of second degree was, the higher was the nonlinearity of regional filling with air in that image pixel. Polynomial coefficients of −0.2 to 0.2 indicated homogeneous regional tidal volume changes during inspiration. Negative values (<−0.2) suggested regional hyperinflation, whereas positive values (>0.2) suggested tidal recruitment. In order to assess the ventral-to-dorsal distribution of regional filling, profiles of average polynomial coefficients were generated in 32 image layers.

Thirdly, changes in EELV were also determined from the EIT data similar to earlier studies [22–24, 29–32]. In brief, the end-expiratory values of Δ*Z* in pixel EIT waveforms depend on regional EELV. An increase in regional end-expiratory lung aeration leads to higher end-expiratory values of Δ*Z,* whereas the opposite happens when EELV falls.

Finally, EIT images were analyzed for the presence of pendelluft as previously reported by others [14]. In summary, the EIT images were divided into four zones (zone 1 and 2 being the non-dependent zone and zone 3 and 4 the dependent zone). The initiation of each breath in each zone was detected; simultaneous inflation of each of the different lung zones was examined to study whether the early inflation in the dependent zone was accompanied by concomitant (transient) deflation of non-dependent zones, indicating movement of gas from non-dependent to dependent lung regions.

All EIT data analyses were carried out using AUSPEX v1.6 (VUmc, Amsterdam, the Netherlands) and MATLAB (MathWorks, Natick, MA, USA).

### Statistical analysis

Normality of data was assessed using the Kolmogorov–Smirnov test. Continuous data were expressed as median and the 25–75% interquartile range (IQR), dichotomous data as percentage (%) of total. The primary endpoint for this study was the CoV. We calculated that we needed to enroll at least *N* = 20 patients to detect equivalence (margin 0.075) with a power of 0.90 and alpha 0.05. Secondary endpoints included lung mechanics, oxygenation index (OI) and relative changes in end-expiratory Δ*Z*. Continuous data were compared between M0 and M1 using the Wilcoxon signed-rank test. Comparisons between two groups were made with the independent samples *T* test or the Mann–Whitney *U* test, dependent upon the distribution of the data. All analyses were performed with SPSS version 22 for Mac OS X (Chicago, Ill, USA). *p* values less than 0.05 were accepted as statistically significant.

## Results

### Patients

A total number of 22 patients were studied. Baseline characteristics are summarized in Table 1. Their median age was 15 weeks (7.8–77.5), and the median weight 5.1 kg (4.2–11.7). The admission diagnosis underlying AHRF was pulmonary in 77.3% of patients. The median day of examination was 2 (1.8–6.3). The population suffered from severe lung injury, signified by a median LIS of 9 (8–10). The mortality rate of the whole study population was 13.6%. Six patients had mild PARDS, and 16 had moderate or severe PARDS. There were no differences in age, PRISM III and admission diagnosis between the two ARDS groups.

**Table 1:** Characteristics of *N* = 22 pediatric patients with acute hypoxemic respiratory failure (AHRF)

Number of patients	*N* = 22
Male/female (%)	45.5/54.5
Age (weeks)	15 (7.8–77.5)
<12 months (%)	68.2
Weight (kg)	5.1 (4.2–11.7)
PRISM III (24 h) score	4 (1–6.5)
Admission diagnosis (%)	
Respiratory	77.3
Postoperative	9.1
Shock	9.1
Renal	4.5
No comorbidities (%)	77.3
Lung injury score	9 (8–10)
PARDS (%)	81.8
Moderate to severe (%)	59.1
Expiratory tidal volume (mL/kg)	7.3 (6.4–8.7)
PEEP (cmH_2_O)	8 (6–10)
FiO_2_	0.62 (0.50–0.81)
Duration of mechanical ventilation (days)	10 (7–16)
Length of PICU stay (days)	13.5 (8–29.5)
Mortality (%)	13.6

Continuous data are expressed as median (25–75 interquartile range), and categorical data are expressed as percentage of total

*PRISM* Pediatric Risk of Mortality Score, *PARDS* pediatric acute respiratory distress syndrome, *PEEP* positive end-expiratory pressure, FiO_2_ fraction of inspired oxygen, *PICU* pediatric intensive care unit

### Effects of NMB on tidal volume distribution and regional lung filling characteristics

The overall median (25–75 IQR) CoV [before 48.8% (46.6–50.3) and during NMB 47.9% (45.1–50.6)] was not significantly affected by NMB. EELV was not significantly changed by the introduction of NMB, suggesting that atelectasis did not occur. This was confirmed by the regional filling characteristics, showing a median polynomial coefficient of −0.05 ± 0.11 (range −0.36 to 0.01). Significant breath-to-breath variation was not observed. Overall, the use of NMB did not affect the distribution of polynomial coefficients, suggesting homogeneous tidal lung inflation. In addition, we did not observe the occurrence of pendelluft in any patient before the introduction of NMB agents.

**Fig. 1:**
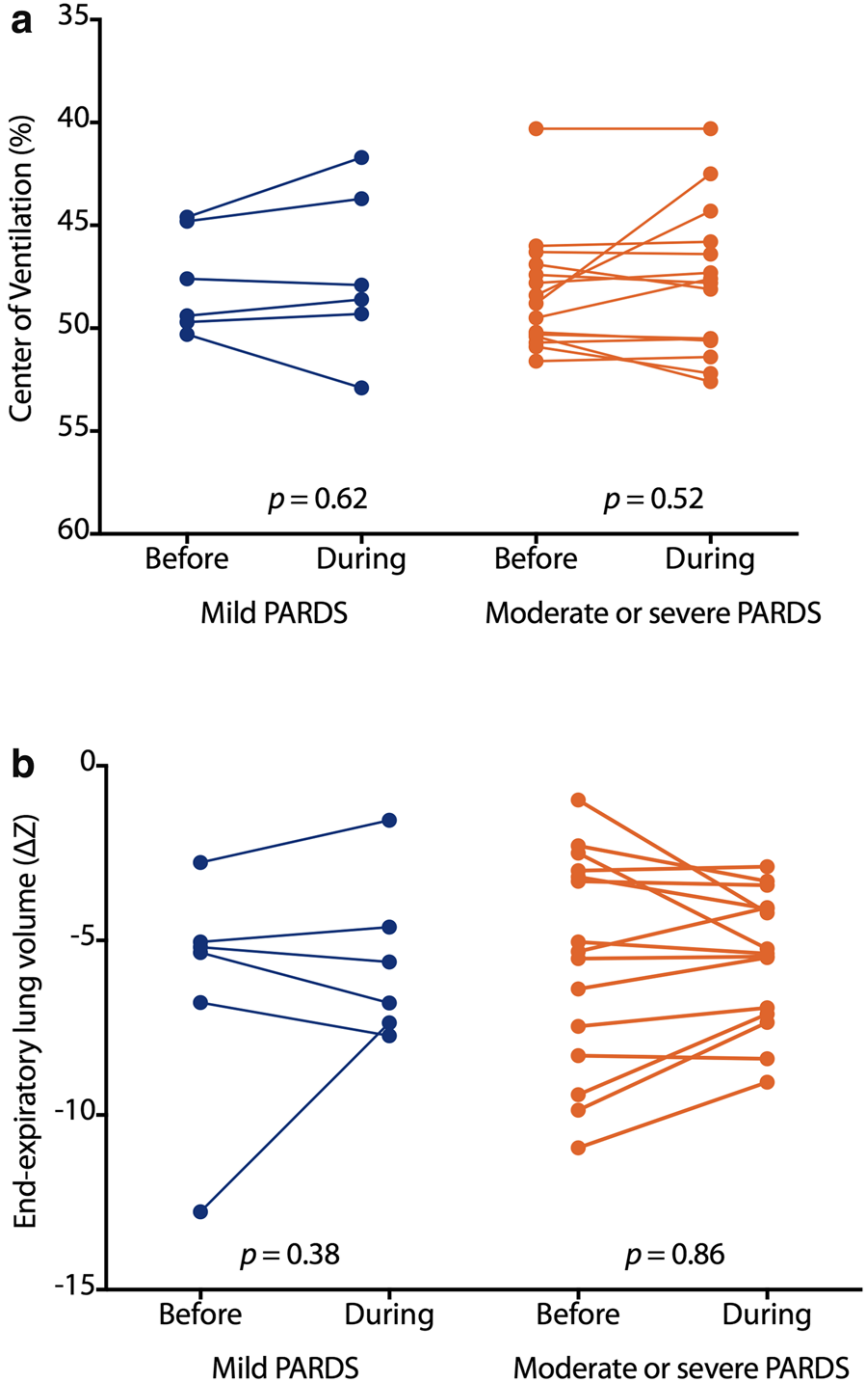
Center of ventilation (CoV) (**a**) and end-expiratory lung volume (EELV) (**b**) measured using electrical impedance tomography (EIT) in *N* = 6 patients with mild pediatric acute respiratory distress syndrome (PARDS) and *N* = 16 patients with moderate or severe PARDS. CoV is expressed as percentage of total with 0% indicating the uppermost ventral position and 100% indicating the lowermost dorsal position. EELV is expressed as median

**Fig. 2:**
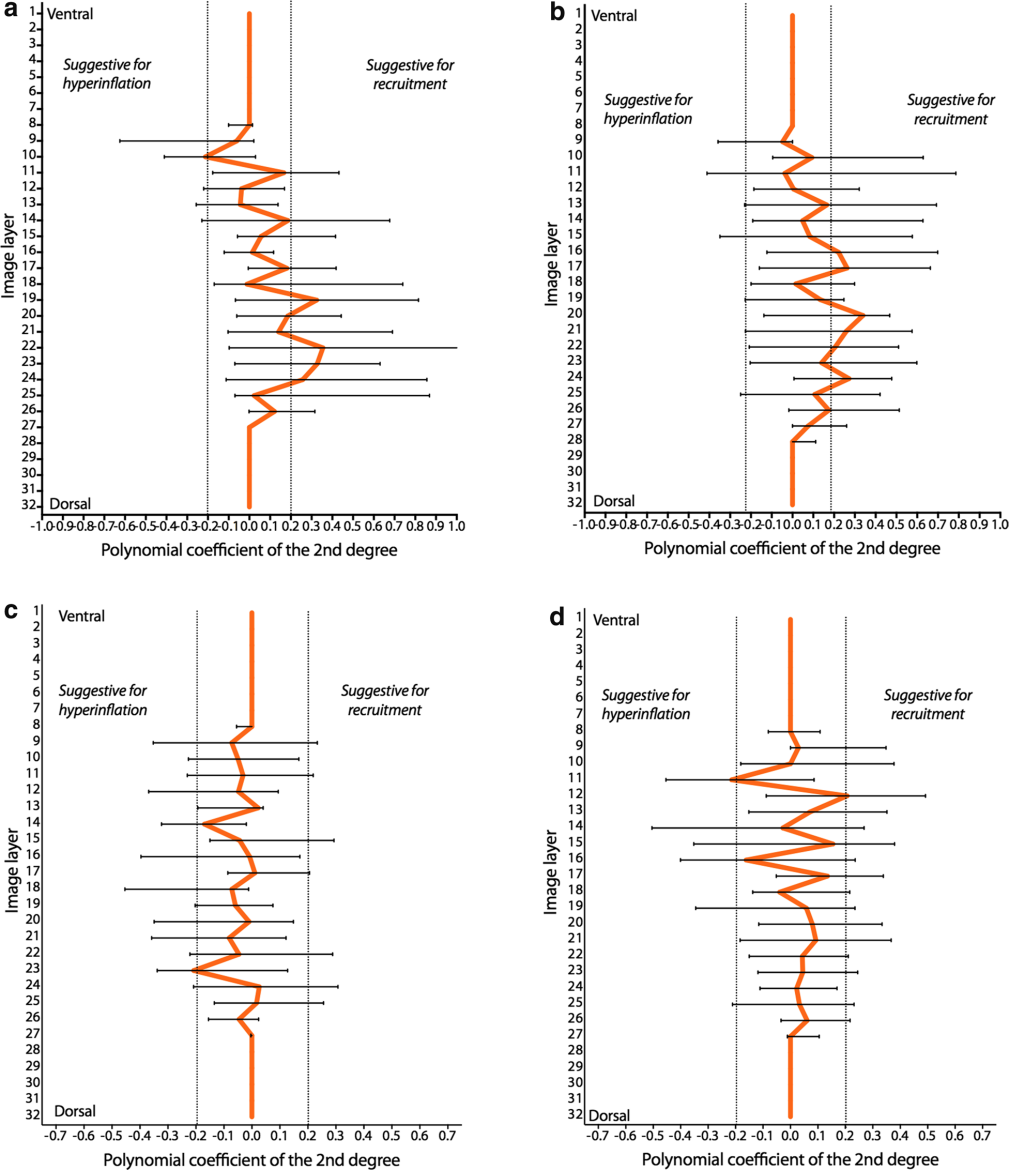
Distribution of polynomial coefficients of regional lung filling characteristics during inspiration in the lung region within 32 anteroposterior image layers measured using electrical impedance tomography (EIT) in *N* = 6 patients with mild pediatric acute respiratory distress syndrome (PARDS) and *N* = 16 patients with moderate or severe PARDS. Data are expressed as median (25–75 interquartile range). Polynomial coefficients less than −0.2 suggest regional hyperinflation, greater than 0.2 regional potential for recruitment and between −0.2 and 0.2 homogenous tidal inflation during inspiration. **a** Mild pediatric ARDS, before neuromuscular blocking agents, **b** mild pediatric ARDS, during neuromuscular blocking agents, **c** moderate or severe pediatric ARDS, before neuromuscular blocking agents, **d** moderate or severe pediatric ARDS, during neuromuscular blocking agents

When comparing patients with mild PARDS with patients classified as moderate or severe PARDS, no significant differences in changes in CoV (Fig. 1a) or EELV (Fig. 1b) were observed. Also, the distribution of polynomial coefficients was not different between the two groups (Fig. 2).

### Effects of NMB on global lung mechanics and indices for oxygenation and ventilation

 Table 2 summarizes the data on lung mechanics, hemodynamics and metrics for oxygenation and gas exchange. A significant decrease in mPaw (*p* = 0.039) occurred in all patients after the introduction of NMB agents [before 14 (13–16), after 14 (13–15)]. There were no significant changes in *P*_plat_ or Crs. The AVDSF increased significantly (*p* < 0.001) after start of NMB agents. Hemodynamics including heart rate [before 155 (123–173) and during NMB 162/min (134–178)] and invasively measured mean arterial blood pressure [before 63 (55–74) and during NMB 60 mmHg (49–71)] did not significantly change between the examination time points M0 and M1. Subgroup analysis showed that the improvement in OI was only significant (*p* = 0.01) in patients with moderate or severe PARDS (*N* = 16). Also, in these patients the PaO_2_/FiO_2_ ratio increased (*p* = 0.016) and the AVDSF increased (*p* = 0.005).

**Table 2:** Data on lung mechanics, hemodynamics and metrics for oxygenation and gas exchange in *N* = 22 pediatric patients with acute hypoxemic respiratory failure (a) all patients and (b) mild versus moderate-to-severe PARDS

	All patients (*N* = 22)	
	Before	During
(a)		
Lung mechanics		
*P*_plat_ (cmH_2_O)	28 (26–33)	29 (26–30)
mPaw (cmH_2_O)	14 (13–16)	14 (13–15)*
Driving pressure (*V*_T_/Crs) (cmH_2_O)	15.7 (11.6–22.9)	16.4 (7.9–19.4)
Plat–PEEP (cmH_2_O)	22 (17–26)	21 (19–23)
PEEP (cmH_2_O)	8 (6–10)	8 (6–10)
Respiratory rate (/min)^a^	40 (34–43)	35 (30–40)*
Compliance (mL/cmH_2_O/kg)	0.51 (0.34–0.77)	0.45 (0.38–0.87)
Resistance (cmH_2_O/L/kg/s)	26.2 (5.5–40.6)	24.5 (6.5–31.3)
Hemodynamics		
Heart rate (/min)	155 (126–170)	162 (137–176)
Mean arterial pressure (mmHg)	63 (56–73)	60 (50–69)
Metrics for oxygenation and gas exchange		
PaO_2_ (mmHg)	65 (58–102)	76 (66–103)
PaCO_2_ (mmHg)	48 (44–56)	51 (44–61)
End-tidal CO_2_ (mmHg)	39 (35–46)	39 (35–46)
pH	7.35 (7.30–7.40)	7.34 (7.22–7.39)
Oxygenation index	12.8 (7.5–16.9)	10.2 (7.8–14.5)*
PaO_2_/FiO_2_ ratio	104 (90–168)	133 (95–172)
SpO_2_/FiO_2_ ratio	150 (115–186)	142 (111–178)
AVDSF	0.19 (0.10–0.28)	0.22 (0.14–0.34)**

	Mild PARDS (*N* = 6)		Moderate or severe PARDS (*N* = 16)	
	Before	During	Before	During
*(b)*				
Lung mechanics				
*P*_plat_ (cmH_2_O)	28 (23–29)	29 (24–32)	30 (26–35)	29 (26–30)
mPaw (cmH_2_O)	13 (10–15)	13 (10–14)	15 (13–16)	14 (13–16)
Driving pressure (*V*_T_/Crs) (cmH_2_O)	18.3 (10.6–26.8)	17.1 (7.1–19.7)	15.0 (10.0–22.6)	18.3 (12.5–21.4)
Plat–PEEP (cmH_2_O)	20 (14–24)	21 (17–26)	22 (17–28)	21 (18–23)
PEEP (cmH_2_O)	6.5 (5–8.5)	6.5 (5–8.5)	8 (7.3–10)	8 (7.3–10)
Respiratory rate (/min)^a^	35 (29–50)	35 (23–46)*	40 (33–43)	38 (30–40)
Compliance (mL/cmH_2_O/kg)	0.66 (0.46–0.98)	1.16 (0.41–1.81)	0.50 (0.31–0.70)	0.45 (0.37–0.82)
Resistance (cmH_2_O/L/kg/s)	15.8 (3.7–47.6)	16.0 (2.9–42.0)	26.2 (8.9–41.3)	24.5 (10.3–31.3)
Hemodynamics				
Heart rate (/min)	152 (113–191)	155 (111–188)	155 (139–163)	162 (140–173)*
Mean arterial pressure (mmHg)	71 (60–76)	63 (53–77)	60 (55–71)	58 (49–69)
Metrics for oxygenation and gas exchange				
PaO_2_ (mmHg)	128 (99–188)	102 (74–216)	62 (56–72)	70 (63–90)*
PaCO_2_ (mmHg)	50 (42–59)	53 (42–65)	45 (44–58)	51 (44–61)
End-tidal CO_2_ (mmHg)	39 (34–49)	41 (31–49)	39 (34–46)	39 (35–44)
pH	7.34 (7.21–7.44)	7.33 (7.25–7.39)	7.36 (7.32–7.40)	7.34 (7.19–7.40)
Oxygenation index	4.5 (4.0–7.2)	6.1 (4.9–7.5)	15.2 (12.2–23.3)	12.5 (10.0–19.1)*
PaO_2_/FiO_2_ ratio	286 (164–337)	207 (151–272)	100 (81–117)	109 (87–154)*
SpO_2_/FiO_2_ ratio	193 (115–246)	165 (121–216)	141 (105–175)	135 (105–177)
AVDSF	0.20 (0.12–0.31)	0.24 (0.14–0.32)	0.17 (0.06–0.26)	0.22 (0.14–0.35)**

Data are expressed as median (25–75 interquartile range)

*PARDS* pediatric acute respiratory distress syndrome, *P*_plat_ plateau pressure, *mPaw* mean airway pressure, *V*_T_ tidal volume measured by the ventilator; respiratory system compliance, *AVDSF* end-tidal alveolar dead space fraction

* *p* < 0.05; ** *p* < 0.01 on paired analysis (before neuromuscular blockade vs during neuromuscular blockade)

^a^Respiratory rate is the machine breaths and spontaneous breaths

## Discussion

This study showed that anteroposterior *V*_T_ distribution and regional lung filling characteristics were not affected by the use of NMB in children with AHRF. There was no evidence for pendelluft during spontaneous breathing. In addition, an immediate improvement in OI, PaO_2_/FiO_2_ and decreased mPaw following the institution of NMB was noted in patients with moderate-to-severe PARDS.

Center of ventilation was not affected in our study, indicating that the delivery of *V*_T_ was not preferentially directed toward the non-dependent zone immediately after initiation of NMB. The location of CoV remained within the same range in the middle parts of the lung plane. This is different from mechanically ventilated adults. In adults without ARDS, absence of spontaneous breathing during MV resulted in alveolar collapse and a preferential delivery of the *V*_T_ to the ventral parts of the lung [33, 34]. Also, we did not observe a gravity-dependent distribution of the polynomial coefficients describing regional filling characteristics of the lungs as has been described in adults with ARDS [21]. This may be explained by the fact that the anteroposterior chest wall dimension is smaller in infants than in adults and the gravity effect is less pronounced. Moreover, the chest wall has a circular shape in early childhood, so it can be speculated that the effects of gravity may be stronger in the middle part where the mediastinum is. Furthermore, lung recoil is lower in younger children. Absence of gravitational effects has previously been described in EIT studies on infants [35, 36]. On the other hand, the EIT image also contains information regarding the chest wall. The chest wall of infants is more compliant than adults, which speculatively could have led to overestimation of the effects of the chest wall (thus collapsed lung zones may have been interpreted as chest wall).

We did not confirm the occurrence of pendelluft during spontaneous breathing such as observed in adult ARDS [14]. There is no easy explanation for this difference between children and adults. Our patient population was very young; this means that interalveolar pores and bronchoalveolar channels may have not been developed yet [37, 38]. This does not rule out, however, the possibility of pendelluft via the airways. On the other hand, the respiratory drive of the patients when they were not paralyzed may have not been enough to cause pendelluft as the median pH approached normal values and all patients received continuous infusion of sedative and analgesic drugs. We have no information on the P0.1 in our patients.

Spontaneous breathing during invasive MV has been advocated for critically ill adults with ARDS as it improves lung mechanics, oxygenation and patient outcome, but the recent ACURASYS trial challenged this assumption [8, 9, 39, 40]. Possible explanations include a decreased ${\dot{\text{V}}}$O_2_ following the institution of NMB. The effect of NMB on a reduction in ${\dot{\text{V}}}$O_2_ after the introduction of NMB is less clear in pediatric patients. Vernon et al. observed a modest reduction in ${\dot{\text{V}}}$O_2_ of 8.7 ± 1.7% and that in energy expenditure of 10.3 ± 1.8% of pre-NMB values in 20 mechanically ventilated children of whom five had acute respiratory failure [41]. Lemson et al. were unable to find any reduction in ${\dot{\text{V}}}$O_2_ in pediatric cardiac surgery patients [42]. We observed a significant improvement in PaO_2_/FiO_2_ in the presence of a fixed FiO_2_ in patients with moderate or severe PARDS that could not be explained by an increase in EELV. Hence, it cannot be ruled out that a reduction in ${\dot{\text{V}}}$O_2_ explains the improvement in OI in this study, but this needs further exploration.

Alternatively, introducing NMB would reduce the mechanical impact of dyssynchrony. We did observe a decrease in mPaw. This is an interesting finding, because mPaw is influenced by PIP, PEEP, respiratory rate and inspiratory time. It is usually measured over a number of (spontaneous) breaths. As expected, there was no significant change in *P*_plat_ and the inspiratory time and number of mandatory breaths were fixed in this study. Hence, it may be surmised that the introduction of NMB indeed reduced the mechanical impact of dyssynchrony by reducing the transpulmonary pressure because patient’s spontaneous effort ceased. This assumption is supported by previous experimental work in lavage-injured rabbits showing increased transpulmonary pressure despite pressure-limited ventilation using moderate *V*_T_ (7–9 mL/kg) in animals with strong spontaneous breathing activity [43]. In particular, in animals with severe lung injury spontaneous breathing worsened lung injury [44]. Future studies including electromyography (EMG) measurements of the diaphragm and other respiratory muscles as well as esophageal manometry are indicated to confirm the effects of NMB on reducing the mechanical impact of dyssynchrony and active expiration in pediatric patients with moderate-to-severe PARDS. As our study primarily had included very young children, future work should also study the effects of NMB in older children with lung and chest wall physiology presumed to be intermediate between infants and adults and also if there is a difference in response to NMB between patients with pulmonary versus non-pulmonary PARDS.

Lung mechanics including *P*_plat_ and Crs were not influenced by NMB in this study. Furthermore, we did not observe a significant decrease in EELV after the introduction of NMB. This may be explained by the short study period as others have shown a decrease in EELV in healthy, anesthetized children immediately after introduction of NMB, which could easily be overcome by the application of PEEP [45, 46]. However, these authors included patients without lung injury and used a multibreath washout technique to measure EELV; hence, our study cannot easily be compared with these earlier observations.

We did observe an increase in AVDSF which could be interpreted as an increase in dead space. This is somewhat contradictory to the finding of an improved PaO_2_/FiO_2_ in the presence of a fixed FiO_2_ which could be contemplated as a proxy for reduced shunt. There is no easy explanation for the increased AVDSF, especially in patients with moderate-to-severe PARDS. As discussed earlier, we did not find a decrease in EELV or changes in regional filling characteristics suggestive for atelectasis, but this cannot be ruled out. It may also be speculated that before introducing NMB spontaneous efforts of the patient contributed to alveolar ventilation. Supportive for this is the observation that pCO_2_ increased after introduction of NMB (Table 2). Future studies are needed examining the effects of NMB on pulmonary circulation stratifying for differences in ARDS severity.

## Strength and limitations

Physiological studies in pediatric patients with ARDS are very limited. The strength of this study is that it is the first to explore the physiological effects of NMB in PARDS as has been recommended by the Pediatric Acute Lung Injury Consensus Collaborative [47]. However, some limitations need to be addressed. First, it is a single-center study with a small sample size, signifying that the findings need to be confirmed by others. Second, the indication for NMB was at the discretion of the attending physician. There was no clinical decision algorithm when to use NMB, although in our unit continuous use NMB is usually started if the peak inspiratory pressures approximate 28–30 cmH_2_O, and the patient is hypoxemic and continuous to show excessive WOB despite adequate sedation and/or analgesia. However, confounding by indication bias cannot be ruled out [48]. Such confounding can only be overcome by randomization. Third, the effects of NMB were studied after 15 min. The benefit of this approach was that ventilator settings remained constant throughout the study period, which reduced the number of possible confounding effects. Nonetheless, the non-immediate effects of NMB need to be studied in the future as well. Also, we were unable to confirm the efficacy of NMB, as train-of-four measurements were unavailable. Fourth, EIT is a noninvasive technique with the potential to monitor regional lung mechanics [49]. An important limitation of EIT is the fact that the images do not necessarily detect ventilation differences in the whole cephalocaudal direction. They show changes in electrical impedance derived from a three-dimensional chest slice because regions out of the electrode plane also affect the measured electrical potential differences and influence the generated images [50]. However, this does not preclude the underestimation of possible ventilation heterogeneity by EIT in remote chest regions. Furthermore, positioning of the EIT electrodes is important when assessing EELV; this should be kept in mind when comparing studies that used other techniques to study EELV such as multibreath washout.

## Conclusions

Institution of NMB in pediatric patients with AHRF did not affect distribution of *V*_T_ and regional lung filling characteristics. It did result in an immediate improvement in the OI. The improvement in OI could not be attributed to improved distribution of ventilation. Future studies in patients with moderate-to-severe PARDS exploring longer-term effects and outcomes of the use NMB are indicated.
